# The Impact of the Economic Corridor on Economic Stability: A Double Mediating Role of Environmental Sustainability and Sustainable Development Under the Exceptional Circumstances of COVID-19

**DOI:** 10.3389/fpsyg.2020.634375

**Published:** 2021-01-25

**Authors:** Haiyan Li, Javaria Hameed, Rafique Ahmed Khuhro, Gadah Albasher, Wedad Alqahtani, Muhammad Waqas Sadiq, Tong Wu

**Affiliations:** ^1^School of Management, Xizang Minzu University, Xianyang, China; ^2^Business School, Liaoning University, Shenyang, China; ^3^Department of Management Sciences, The University of Haripur, Haripur, Pakistan; ^4^Department of Zoology, College of Sciences, King Saud University, Riyadh, Saudi Arabia; ^5^Department of Management Sciences, COMSATS University Islamabad, Sahiwal, Pakistan; ^6^School of Business, Nanjing University, Nanjing, China

**Keywords:** COVID-19, CPEC project, economic stability, environmental sustainability, sustainable development

## Abstract

This study discusses the impact of different economic indicators on economic stability, including honest leadership, improved infrastructure, revenue generation, and CPEC taking into account the double mediating role of environmental sustainability and sustainable development, while considering the latest COVID-19 situation. This study adopted primary data collection methods and obtained data from the employees of CPEC by using questionnaires and smart-PLS for analysis purposes. The results revealed that honest leadership, improved infrastructure, revenue generation, and CPEC have a positive nexus with economic stability. Despite the severe impact of COVID-19 on the country’s economy, the economic corridor plays a vital role in stabilizing the state’s economy and supports all those related to this phenomenal project either directly or indirectly.

## Introduction

Corridors are important for managers and consumers and play a vital role in bringing economic and social development (Muro-Rodríguez et al., 2017). Considering this importance, China and Pakistan have started a new mega project named the China Pakistan Economic Corridor (CPEC). This project is considered a new hope for a secure future. This project makes trade and transportation of goods easy for Chinese people, and it provides a lot of benefits in terms of technology and infrastructure development. CPEC was declared as a new and innovative venture in the year 2003. This project is a part of China’s One Belt One Road (OBOR) initiative and was started by the Chinese government in 2013. This project is focused on the connectivity of different continents like Europe, Africa, and Asia. CPEC is considered a small part of OBOR (Ibragimov et al., 2019).

It is still regarded as the most extensive project between two immediate neighbors, i.e., Pakistan and China (Haraguchi et al., 2019). CPEC is not a single project, but it is a nexus of a colossal number of developmental projects. Pakistan is a developing country. This country faces many problems like lack of resources, increased taxes, foreign debts, political leader corruption, and economic crisis. These problems have incentivized government authorities. The government of Pakistan is trying to improve the current situation by utilizing native resources (Tarofder et al., 2019). Pakistan’s economic condition was not stable previously, and that is why policymakers and technocrats have advised the government authorities to promote collaboration with different neighboring countries. Other countries with sound investment plans can be proven as the right investment partners for Pakistan (Daniëls et al., 2019).

Pakistan is blessed with many natural resources, while China has started exploring all of Pakistan’s resources. The problems of illiteracy, and social and political pressures had created hurdles in the way of Pakistan’s prosperity (Huang et al., 2017; Alvesson and Einola, 2019). Nowadays, Chinese and Pakistani professionals are trying to devise new ways to explore novel resources. Unskillful and illiterate labor also affects the progress of many projects. Technological advancement and different automated machines are an unknown to the inhabitants of these areas (Asif and Ling, 2019; Garfield et al., 2019). The literacy rate of the inhabitants of these hilly areas near Gwadar port and Gilgit is relatively low. The government should establish skill development institutions and IT firms to assist in the economic and social development of these areas. The unity and cooperation between the Chinese and Pakistani populations is evident in many areas. Chinese people are amicable, and they indulge themselves in developing CPEC and related projects in Pakistan. The new bonding experience between ordinary people of both countries increases daily (Yu, 2017; Hassan, 2020).

China Pakistan Economic Corridor consists of multi developmental projects that are foreseen as the prospects for Pakistan and China’s success. The Chinese government has conceptualized CPEC as a dream of success, and Pakistan is materializing this dream as a bright future. The local Pakistani community is also delighted and excited about this project (Badawi et al., 2019). The educational reforms, infrastructure development, revenue generation, and technological advancements have improved the local community’s lifestyle. The past few years were dreadful and fearful for the residents of Khyber Pakhtunkhwa and Balochistan, Pakistan. That is why some local people were against Chinese intervention and entrance into their local territory. Security-related issues were faced initially by the government of Pakistan. A proper education and awareness-related campaign was started in those areas to educate people about the benefits of the China/Pakistan friendship and CPEC. This education and awareness campaign has brought forth excellent outcomes. The developmental activities like educational institutions, skill development institutions, and modernized infrastructure have reshaped people’s lifestyles (Hussain, 2019).

The economy’s productive base includes its capital assets (produced, human, and natural capital stocks; knowledge) and its institutions (including its cultural coordinates). Together, they supply citizens with the infrastructure they create, use, and exchange. The productive foundation of a culture is the root of its wellbeing. It should be remembered that there is a broad range of resilient items, specific tangibles like buildings and equipment, land and wildlife, trees, and shrubs. While some intangible things like patentable ideas, legal system cultural coordinates, and human resources with shared aspirations need to be considered (Dasgupta, 2007; Awais et al., 2019).

Scope, complexity, and urgency differentiate environmental sustainability. Firstly, the sense of sustainability spans the fields of culture, society, and people to cover the natural environment: air, soil, water, etc. This broadened focus has many consequences, such as environmental performance convergence and the need for innovative testing methodologies and indicators. Second, phenomena of sustainability are dynamic and multi-layered, sometimes distinguished by unclear interdependencies and non-linearity. The dilemma includes alternate frames including logical ones (economic factors such as competitiveness and profitability), ecological ones (environmental protection, including management of natural resources, and climate mitigation), humanistic ones, i.e., personal fulfillment and societal interests such as equal trading policies and ideal rights (Wright, 2002; Martins et al., 2010).

China has a tremendous think tank, and its government has always supported Pakistan. This friendship is growing stronger day by day. The development of the economy of Pakistan is due to the honest efforts of China. In 2013, the Chinese president proposed the Silk Road Economic belt to connect the Baltic Sea with the Pacific Ocean. This project was the necessary foundation of CPEC and the economic stability of Pakistan. It gave them a thought to construct new infrastructure and buildings to provide a *trans-*Eurasian passage for trade. Pakistan is the center point of this passage. That is why China considers CPEC as a vital pathway of trading and economic stability for all Eurasia countries (Jia, 2017; Rahman, 2018; Saud, 2018).

The importance of CPEC and related projects have gained the focus of a lot of other countries like Saudi Arabia. The primary contribution of CPEC is that it enhanced the local public’s assistance in developing the country’s economy and infrastructure. The reforms regarding the revenue generation for the wellbeing of the nation’s economy is enormous. Pakistan and China both have some common traditions and business interests. They both have the same strategic positions and face combined threats with great bravery and brotherhood. The recent technological advancements in the police force and army of both countries have also eliminated the security-related risks of the countries and their adjacent neighbors (Ahmed et al., 2018).

Forensic experts have devised many new techniques to resolve terrorist-related concerns of that area. The Pakistani government has provided many police resources and the forensic branch closely checks all activities related to militancy and terrorist activities in this region. They have ensured the security of all Chinese professionals in the country on an exceptional basis and have properly secured apartments in Pakistan. They have provided proper food and water resources for their foreign friends, and hence the economy and revenue-related departments are also blooming under CPEC and related projects.

While considering all the above, it is also imperative to consider natural disasters that could harm any project’s applicability. Either this project is executed on a significant scale or a minor scale. The same is the case with the current COVID-19 situation. Nobody could think that a single virus would be powerful enough to jeopardize the entire world’s economy so disastrously. That is why this study bears significant importance in researchers’ and other stakeholders’ eyes that either this mega project sustains under this extreme pandemic situation and supports the economy of concerned and relevant countries or collapses on its foundations while fighting with this unexpected natural disaster.

## Literature Review

A moderate amount of research is being conducted by Pakistani and Chinese researchers such as Abid and Ashfaq (2015) who discuss CPEC challenges and opportunities, Ali et al. (2018) who found an energy crises bottleneck and suggested solutions, Chen et al. (2018) who show concerns on heavy investments, Khuhro et al. (2019) who investigated the readiness for organizational change due to CPEC, and Sun et al. (2020) who talked about socio-cultural impacts of CPEC on community wellbeing. This social wellbeing is the result of honest and diligent Chinese leadership that has provided Pakistan with ample working resources and funding in terms of business, employment, and working opportunities. CPEC is a project that will benefit future generations. It will provide the Chinese and Pakistani public with the most effective platform for their countries’ development and prosperity (Makhdoom et al., 2018). The proper regulatory measures are necessary to gauge the efficacy of all working and planning initiatives. A regulatory task force must be designed by combining the skilled personnel of both Chinese and Pakistani origins who will effectively audit all budgeting and fund-related matters. They have to give monthly, and yearly audit reports to the government so that both governments are well aware of the project’s pace. The social media advertisement of CPEC and related projects is necessary to spread the knowledge to Chinese and Pakistani natives and the international audience so that budgeting and investment-related areas can flourish more effectively.

### Hegemonic Stability Theory

In the previous period, the economy’s position was reduced, and countries mostly worked on their military might. Ties with other countries relied on the army itself and their friends and allies on the military force. But the market has now become an essential instrument for developing a non-military formidable force. Countries with economic power may be using a carrot and stick method to exert leverage over trade, financing, aid, grants, and impacts on poorer nations. Rising economic interdependence triggered the critical factor of hard power in the economy (Ahmed, 2019; Hao et al., 2020; Hassan, 2020).

Following these national interest changes, this research work considers the Hegemonic stability theory that is based on international relations and is rooted in the field of economics. This theory argues that economic openness brings stability. Apart from criticism on Hegemonic theory, this theory is widely considered due to its two versions, the first one discusses stability due to a single dominant state, while, the second explains that by taking insights from game theory that logic of collective good brings economic stability (Webb and Krasner, 1989). CPEC is a win-win project between two allied countries that is beneficial for both countries. This research work considers the second Hegemonic stability approach and all hypothesis are build by considering this logic.

### Honest Leadership and Economic Stability

Pakistan is currently facing leadership issues. The two previous ruling parties and their principal leaders were proven to be the most corrupt leaders of the world. They have looted the resources and financial budget of Pakistan. They destroyed the country’s economic stability and devastated Pakistan’s international reputation and its native business community. The foreign investment and stock exchange trends dropped drastically after the proclamation of their corruption in colossal money laundering scandals (Ali et al., 2018; Khan et al., 2020). Chinese leaders are quite diligent and honest in this regard. China is the second-largest economy globally, and it is all due to its leaders and the public’s efforts. The social reforms and efficient justice procedure implementation are necessary for the betterment of all developmental projects.

China Pakistan Economic Corridor is making remarkable progress in this government’s tenure as the regulatory authorities have been quite active in this period. Proper auditing and honest, enthusiastic leadership will surely enhance and support Pakistan and China’s economic stability. Sincere and devoted leaders make the country stable and provide investment and funding opportunities from overseas (Ali et al., 2018). Honest leadership plays an essential role in economic stability. Real leadership will never use short cuts. Simultaneously, dishonest leadership will destroy any country economically because a fraudulent leader’s purpose will be to earn money at any cost in any circumstances. Corruption is another factor that could be damaging to any economy (Khan et al., 2020).

Dishonorable leadership would negatively use their power (Hussain, 2019; Syed, 2020). For example, wrong or substandard proposals would be approved for personal gain that could harm the economy. At the same time, honest leadership would believe in gradual economic progress.

H1: There is a significant positive association between honest leadership and economic stability in the country.

### Improved Infrastructure and Economic Stability

The economy will cover factors such as manufacturing ability and technologies for other entities. Thus, it eventually takes over the military as a significant factor in its control of other countries or states. No country can sustain its military power by investing a lot of capital, resulting from a stable economy. Even if the nation is geographically fine, there is a considerable reserve of natural resources and broad military forces; however, it cannot sustain without a healthy economy (Ahmed et al., 2018).

Infrastructure development is related to a modern, well-developed information technology structure while also considering modern marketing and developmental plans (Sadiq et al., 2020a). The current and well-established routes will provide efficient and advanced transportation facilities to the trading companies and business community (Rimovna and Gennadievna, 2019). The investors become happy when they do not suffer from any loss caused by product damage. The creation of modernized and digitalized police stations and other regulatory authority headquarters also prohibits illegal smuggling practices. These things provide considerable profits to the local business community and international investors (Shabbir and Zeb, 2019). The revolutionized infrastructure and related aspects have provided economic stability to Pakistan and China.

Infrastructure is important for economic stability. If roads are available in a country, things could be sent to market quickly, and the state will grow economically. Similarly, if public transport is easily accessed, then the economy will grow. The working environment is also essential for economic growth. If the working environment is right, then people will do a large amount of work in a short time. And if the working environment is not correct, people will do less work in the same time frame (Garfield et al., 2019; Sadiq et al., 2020b). Facilities also play a crucial role in economic stability. If the people are facilitated, then this will definitely be the cause of economic stability. Otherwise, it can create an economic depression. A competitive environment is also essential in financial stability. In a competitive environment, any country’s economy will grow within a short time. Airports are also a necessary part of economic stability. Through an effective supply chain of transportation and planes, deliveries will be at their highest and will be sent from one destination to another within a short time. Transport is also critical in economic stability. Through transportation, items will be moved from one city to another easily within time. Fair and handsome wages are also significant for economic stability. If an employer pays attractive salaries to their employees, they will be interested in working, and business and the market will grow. Equal opportunities also play an essential role in economic stability. The workers will want to work in a competitive environment (Mehar, 2017; Rehman et al., 2018).

H2: There is a significant positive association between improved infrastructure and economic stability in the country.

### Revenue Generation and Economic Stability

The increased number of tourist visits to northern and hilly areas of Pakistan has provided a lot of revenue to Pakistan, which boosts the economy into becoming more progressive and stable (Kokot et al., 2020). The modernized and digitalized system of tourist resorts and other recreational areas helped a lot in developing and portraying a positive image of Pakistan to the whole world (Šušić et al., 2019). In the previous year, Pakistan’s economy and peace have been sabotaged by terrorism and other problems. The involvement of Chinese visitors and the government has changed this situation altogether. The improved revenue generation has made remarkable progressive outcomes to support Pakistan’s economy (Hardy et al., 2019). China has advertised Pakistani tourist destinations and made them more attractive for foreigners to visit them without any security-related concerns.

H3: There is a significant positive association between revenue generation and economic stability in the country.

### CPEC and Economic Stability

China Pakistan Economic Corridor acts as a nexus of hope and development for Pakistan’s weak economy. Chinese support and efforts are remarkable in this regard. The involvement of Saudi Arabia helped improve Pakistan’s economy (Beg et al., 2019). CPEC brings support for the economy and the trading, education, and IT-related areas of Pakistan. The bonding between the two countries has proven that CPEC has a strong bond to the world’s economy and business communities (Shahzad and Javaid, 2020; Sharma et al., 2020). Many contributions have been enumerated through the development of CPEC; therefore, boosted economies have a broad impact over CPEC contributions. Developed economies significantly invest in projects to gather higher returns or attain various benefits that have essential aspects for boosted economies and people’s living standards (Stanley and Zhang, 2020). Improved living standards have provided people with confidence in their country’s government (Tipene-Leach and Abel, 2019). A significant boost is linked with Pakistan’s economy while emphasizing the cordial ties of CPEC, contributing to Pakistan’s development and economic growth (van Laar et al., 2019). Boosted economies of various developed countries significantly influence countries’ projects with the eminent significance of elements (Khan et al., 2016). Where the economies have been boosted from the projects with international importance, the countries could positively spend all possible amenities that the government retains for its public (Kusdibyo et al., 2019).

Economic relations are mostly dependent on projects that significantly impact countries (Kakar and Khan, 2020). Therefore, countries with strong connections due to joint projects could quickly develop strong relations to counter international pressures. Relationships between countries due to similar projects or collective investments could develop into a friendly relationship and develop economic ties. Countries’ interests are primary aims; therefore, in CPEC, national and economic interests are more dominated by the elaboration of various initiatives. At the same time, they emphasize the projects that have significant importance, and relations between countries and their native people (Saad et al., 2020). CPEC ventures include educational institutes, companies, housing, work openings, and how these ventures will improve their future and help the community (Cole et al., 2020). The results of this research study would show that policymakers and officials are receiving support from local citizens for the CPEC project and therefore create policies to take local citizen advantages into account in future projects. Therefore, based on literature and past research, we establish a model representing the resident’s expectations and advantages of CPEC construction projects (Hao et al., 2020).

H4: There is a significant positive association between China Pakistan Economic Corridor (CPEC) and economic stability in the country.

### Sustainable Development

Sustainable development is a combination of fiscal, environmental, and social aspects. Sustainable development is defined by the World Wildlife Fund-WWF for Nature in 1993 as “the enhancement of human quality of life in the capacity to sustain ecosystems” (Goodland, 1995; Awais et al., 2019). The World Environment and Development Commission (hereinafter WCED) says: “[we need] development which reacts to the requirements of the present era generation without sacrificing the future’s capacity to fulfill its requirements.” As a controlling norm for economic, social, and environmental development, the United Nations adopts sustainability as a way to satisfy requirements to meet its needs without giving potential cycles and to encourage “impartial redistribution of natural costs and financial changes within and across countries” (Zhang et al., 2017). According to WCED, sustainability includes the strengthening of environmental and natural infrastructure and the provision of current and prospective ancestors’ economic and social welfare. Sustainability has been recognized as the critical regulatory criterion and standard for a modern society to have an expanded normative association between current and potential cohorts. Sustainability is a centralizing idea that represents ecological, environmental, and social aspects as three elementary aspects, meaning the pillars of sustainability need to contemplate human, economic, and natural resources (Kajikawa, 2008; Schoolman et al., 2012). This research seeks to examine the critical foundations of sustainability and address synergies for the essential purpose of sustainable growth. The three facets of sustainability undertaken may have both beneficial and detrimental consequences. In the course of community theses, synergies were revealed and researched and recognized for local economic income by economic tourism, such as scenic scenery, biodiversity, and a high environmental standard, from which neighborhoods would benefit (Gurung and Scholz, 2008).

H5: Sustainable development positively mediates the positive association between honest leadership and economic stability in the country.

H6: Sustainable development positively mediates the association between improved infrastructure and economic stability in the country.

H7: Sustainable development positively mediates the association between revenue generation and economic stability in the country.

H8: Sustainable development positively mediates the association between China Pakistan Economic Corridor (CPEC) and economic stability in the country.

### Environmental Sustainability

The researchers certainly embodied the idea of economic sustainability (Ali, 2018). There is a need to preserve the environment/nature from uncontrolled expansion before it is too late, we must protect human wellbeing (Awais et al., 2019). The means of development in many areas of the world intensify why groundwater, fishing, tropical forests, top-soil, and biodiversity are collapsing and dispersing their once-existing patrimony. The rapid loss of vital resources, air quality, and land destruction confirm that human industrial activities are significantly disrupting global environmental support and that potential biophysical transport capacity is more likely to be reduced (Zhang et al., 2017). The foreign allocation of investments abroad promotes economic development and shifts its environmental threats to the host countries. For emerging technology that is less energy-intensive and for renewable resources, additional financial help, and sustainable development paths are needed (Ali, 2018; Awais et al., 2019). Northern Pakistan (NP) is host to three of the world’s largest glaciers. There are 5218 square miles (approx. 15,040 km2) of glaciers and snow covers 2738 square miles. Unfortunately, these glaciers disappear day by day because of several global changes and anthropogenic actions (Gilany and Iqbal, 2016). Due to the CPEC project, nearly 7,000 trucks will travel through this region every day and emit more than 36.5 million tons of CO_2_. This release would relentlessly minimize the glaciers and contribute to extreme water overflow (Laghari, 2013). Also, several businesses would be seriously impacted in some places due to the water crisis, including irrigation, food production for the rising population, hydropower generations, and water-based operational industries. A degraded natural climate, massive glacial scaling, and shifts in the patterns caused by massive CPEC travel will damage biodiversity (Nabi et al., 2017). Aquatic biodiversity on the coastline of the Gwadar Port, the Arab Sea, and Pakistan will be greatly influenced. The areas beside the CPEC routes are particularly vulnerable to climate change owing to the recent global warming patterns (Asmat et al., 2018).

H9: Environmental sustainability positively mediates the positive association between honest leadership and economic stability in the country.

H10: Environmental sustainability positively mediates the association between improved infrastructure and economic stability in the country.

H11: Environmental sustainability positively mediates the association between revenue generation and economic stability in the country.

H12: Environmental sustainability positively mediates the association between China Pakistan Economic Corridor (CPEC) and economic stability in the country.

## Research Methods

For the high rate of economic growth, stability in economic conditions is needed. CPEC plays a vital role in the economic stability in both China and Pakistan. This study highlights the double mediating role of environmental sustainability and sustainable development between honest leadership, improved infrastructure, revenue generation, CPEC, and economic stability.

This study uses a quantitative method to analyze the data acquired and examine the proposed hypotheses’ accuracy. The data for our research have been obtained from the officials and employees involved in the CPEC project. Simple random sampling has been applied to collect relevant data and analyze the data, and for the validity of hypotheses, smart PLS has been adopted. The required data supporting our study have been acquired by distributing questionnaires among officials and employees involved in CPEC. For this study, 570 questionnaires were sent to the target employees and officials. Out of which, 354 were returned, meaning that 62.11% of the targets responded.

**FIGURE 1:**
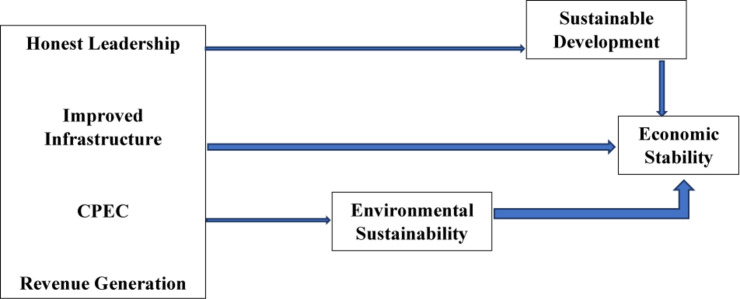
Theoretical model.

The study has four economic stability indicators: honest leadership, improved infrastructure, CPEC, and revenue generation with different questionnaire items. The honest leadership (HLS) section has four items (Abdullah et al., 2016) in relation to economic stability while improved infrastructure (IIFS) has six items (Chuang and Lin, 2013). Revenue generation (RG) also has six items (Rust et al., 2002) and CPEC is comprised of five items (Saad et al., 2019). Besides, the dependent variable in this study, which is the economic stability, has five items. The study has two mediators named as environmental sustainability (ES), which has six items (Green et al., 2019), and sustainable development (SD), which has four items (Kushwaha and Sharma, 2016). All these indicators are shown with the help of Figure 1.

**FIGURE 2:**
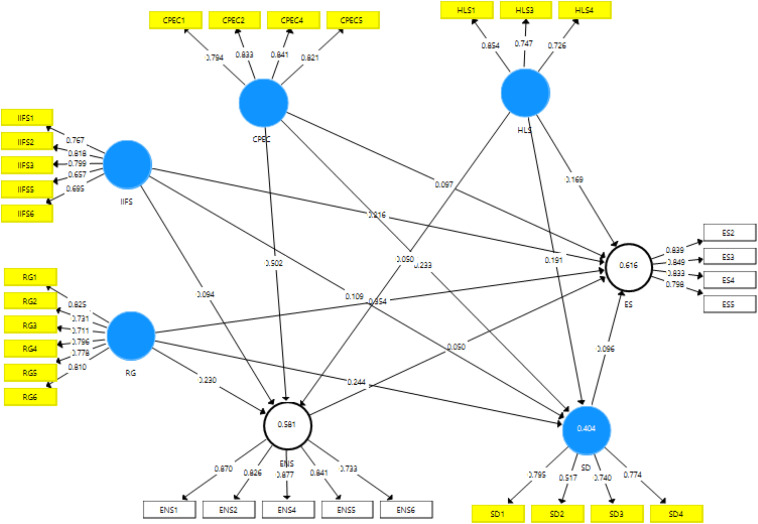
Measurement model assessment.

The outcomes of the study show the assessment of structural and measurement model (Figure 2). Firstly, the measurement model’s assessment has been executed, and convergent validity is part of the measurement model assessment. It has been checked first for the correlation between items. The results indicated that there was valid convergent validity and the extensive correlation between items because the values of Alpha along with CR were across the standard of 0.70; on the other hand, the values of loadings and CR were higher than 0.50. These figures are shown in Table 1.

**TABLE 1:** Convergent validity.

**Constructs**	**Items**	**Loadings**	**Alpha**	**CR**	**AVE**
CPEC	CPEC1	0.794	0.840	0.893	0.676
	CPEC2	0.833			
	CPEC4	0.841			
	CPEC5	0.821			
Environmental sustainability	ENS1	0.870	0.887	0.918	0.691
	ENS2	0.826			
	ENS4	0.877			
	ENS5	0.841			
	ENS6	0.733			
Economic stability	ES2	0.839	0.849	0.898	0.689
	ES3	0.849			
	ES4	0.833			
	ES5	0.798			
Honest leadership	HLS1	0.854	0.775	0.820	0.605
	HLS3	0.747			
	HLS4	0.726			
Improved infrastructure	IIFS1	0.767	0.802	0.864	0.562
	IIFS2	0.818			
	IIFS3	0.799			
	IIFS5	0.657			
	IIFS6	0.695			
Revenue generation	RG1	0.825	0.869	0.901	0.602
	RG2	0.731			
	RG3	0.711			
	RG4	0.796			
	RG5	0.778			
	RG6	0.810			
Sustainable development	SD1	0.795	0.773	0.803	0.511
	SD2	0.517			
	SD3	0.740			
	SD4	0.774			

Secondly, the measurement model assessment was executed with the help of discriminant validity for the correlation between variables. Fornell-Larcker and cross-loadings were used to check the discriminant validity. The results indicated that there was valid discriminant validity and low correlation between variables because the values that show association with the construct itself were larger than the values exposed to the association with other constructs. These figures are highlighted in Tables 2, 3.

**TABLE 2:** Fornell-Larcker.

	**CPEC**	**ENS**	**ES**	**HLS**	**IIFS**	**RG**	**SD**
CPEC	0.822						
ENS	0.726	0.831					
ES	0.622	0.586	0.830				
HLS	0.572	0.484	0.551	0.778			
IIFS	0.542	0.519	0.611	0.411	0.750		
RG	0.628	0.623	0.702	0.472	0.574	0.776	
SD	0.555	0.537	0.548	0.484	0.454	0.543	0.715

**TABLE 3:** Cross-loadings.

	**CPEC**	**ENS**	**ES**	**HLS**	**IIFS**	**RG**	**SD**
CPEC1	**0.794**	0.571	0.522	0.332	0.427	0.505	0.451
CPEC2	**0.833**	0.589	0.553	0.365	0.441	0.555	0.441
CPEC4	**0.841**	0.634	0.486	0.563	0.468	0.491	0.477
CPEC5	**0.821**	0.593	0.487	0.622	0.447	0.515	0.455
ENS1	0.600	**0.870**	0.441	0.418	0.408	0.432	0.481
ENS2	0.593	**0.826**	0.563	0.342	0.462	0.585	0.414
ENS4	0.668	**0.877**	0.478	0.482	0.430	0.510	0.492
ENS5	0.673	**0.841**	0.473	0.447	0.466	0.472	0.502
ENS6	0.466	**0.733**	0.472	0.317	0.380	0.588	0.332
ES2	0.552	0.521	**0.839**	0.554	0.519	0.554	0.422
ES3	0.494	0.455	**0.849**	0.410	0.514	0.622	0.484
ES4	0.501	0.463	**0.833**	0.392	0.534	0.602	0.469
ES5	0.519	0.508	**0.798**	0.472	0.458	0.552	0.445
HLS1	0.564	0.451	0.505	**0.854**	0.386	0.441	0.471
HLS3	0.331	0.279	0.369	**0.747**	0.235	0.273	0.319
HLS4	0.403	0.377	0.393	**0.726**	0.317	0.364	0.314
IIFS1	0.391	0.327	0.432	0.297	**0.767**	0.452	0.343
IIFS2	0.416	0.361	0.486	0.290	**0.818**	0.514	0.377
IIFS3	0.379	0.320	0.515	0.310	**0.799**	0.473	0.353
IIFS5	0.383	0.437	0.396	0.316	**0.657**	0.337	0.281
IIFS6	0.452	0.488	0.448	0.322	**0.695**	0.367	0.339
RG1	0.506	0.489	0.668	0.395	0.482	**0.825**	0.547
RG2	0.576	0.594	0.630	0.409	0.520	**0.731**	0.413
RG3	0.368	0.428	0.557	0.400	0.347	**0.711**	0.442
RG4	0.493	0.470	0.448	0.331	0.454	**0.796**	0.354
RG5	0.483	0.445	0.416	0.340	0.414	**0.778**	0.348
RG6	0.473	0.438	0.468	0.293	0.426	**0.810**	0.374
SD1	0.418	0.385	0.417	0.389	0.404	0.400	**0.795**
SD2	0.309	0.242	0.273	0.264	0.229	0.279	**0.717**
SD3	0.482	0.486	0.486	0.365	0.342	0.476	**0.740**
SD4	0.346	0.377	0.351	0.350	0.297	0.362	**0.774**

*The values are related to HTMT analysis which is used to analyze discriminant validity of items. The values in bold highlight that these values are greater than the values which appear after them, hence proving that these items fulfill the criteria of discriminant validity.*

Secondly, the Heterotrait-Monotrait (HTMT) ratio was used for checking the discriminant validity, and the results indicated that there was valid discriminant validity and low correlation between variables because the values were less than 0.90. These figures are highlighted in Table 4.

**TABLE 4:** Heterotrait-Monotrait ratio.

	**CPEC**	**ENS**	**ES**	**HLS**	**IIFS**	**RG**	**SD**
CPEC							
ENS	0.838						
ES	0.737	0.675					
HLS	0.737	0.611	0.716				
IIFS	0.659	0.612	0.738	0.545			
RG	0.728	0.701	0.796	0.593	0.679		
SD	0.727	0.677	0.711	0.697	0.607	0.684	

The structural model assessment was made using path analysis. The results revealed that honest leadership, improved infrastructure, revenue generation, and CPEC had a positive nexus with economic stability and accepted H1, H2, H3, and H4. The outcomes also revealed that sustainable development positively mediated among the nexus of honest leadership, improved infrastructure, revenue generation, CPEC, and economic stability and accepted H5, H6, H7, and H8. Finally, the outcomes also revealed that environmental sustainability positively mediated among the nexus of honest leadership, improved infrastructure, revenue generation, CPEC, and economic stability and accepted H9, H10, H11, and H12. These figures are shown in Table 5.

**TABLE 5:** Path analysis.

**Relationships**	**Beta**	**S.D.**	***t*-statistics**	***p*-values**	**L.L.**	**UL.**
CPEC - > ES	0.097	0.038	2.560	0.012	0.032	0.167
HLS - > ES	0.169	0.028	5.954	0.000	0.112	0.220
IIFS - > ES	0.216	0.032	6.851	0.000	0.156	0.276
RG - > ES	0.354	0.033	10.612	0.000	0.289	0.417
CPEC - > ENS - > ES	0.125	0.050	2.500	0.041	0.015	0.055
HLS - > ENS - > ES	0.102	0.043	2.372	0.039	0.001	0.028
IIFS - > ENS - > ES	0.105	0.044	2.386	0.044	0.002	0.041
RG - > ENS - > ES	0.111	0.049	2.265	0.038	0.007	0.026
CPEC - > SD - > ES	0.022	0.008	2.692	0.008	0.006	0.040
HLS - > SD - > ES	0.018	0.008	2.412	0.018	0.006	0.034
IIFS - > SD - > ES	0.010	0.005	2.036	0.044	0.004	0.023
RG - > SD - > ES	0.023	0.008	2.946	0.004	0.009	0.037

**FIGURE 3:**
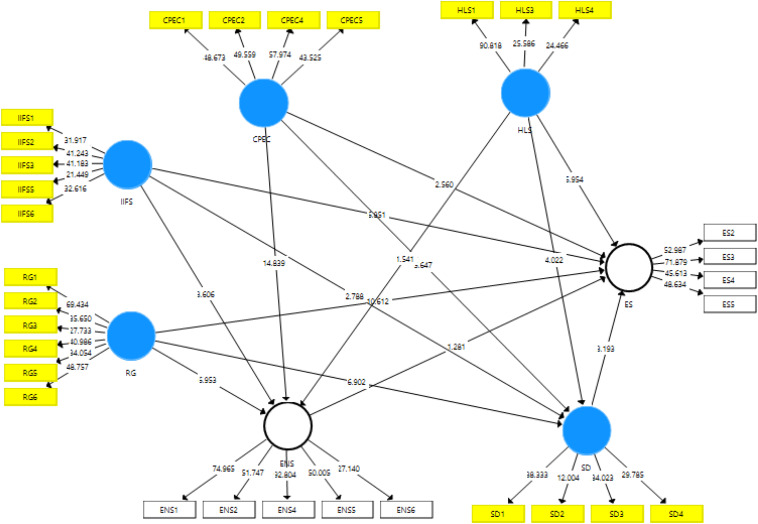
Structural model assessment.

Figure 3 depicts the assessment of the structural model which has seven variables, and each variable has its items with regression weights. All variables and item regression weights are up to standard and follow statistical considerations.

## Discussion and Implication

The study results have revealed that honest leadership in business has a positive relationship with the economy’s stability. These results are in line with the past studies of Puaschunder (2017). It has been proven that an honest leadership in business enterprises in an economy brings stability in the economic conditions and growth. The results have revealed that improved infrastructure has a positive effect on economic stability. These results match with those of other studies (Mohmand et al., 2017) according to which the application of higher quality infrastructure in economic activities brings stability into the economy. The results have indicated that the revenue generation rate has a positive influence on the country’s economic stability. These results agree with the studies of Yan (2011), where the business enterprises that have better earnings show a considerable contribution to economic stability. This paper’s findings have represented that CPEC has a significant impact on economic stability. These results are in line with previous studies (Hassan, 2020) according to which the CPEC project strengthens the countries’ economic stability. The results have indicated that environmental sustainability is an appropriate mediator between CPEC project factors and economic stability. These results agree with previous studies (Ahmed, 2019) proving the same point. The results have also indicated that sustainable development is an essential mediator between CPEC project factors and economic stability. These results are in line with past studies (Khan et al., 2020) which prove that sustainable development strengthens the links between CPEC projects and economic stability factors.

From the perspective of theoretical implications, this study contributes to the literature on the economy. This study begins to describe the influences of honest leadership in business enterprises, the application of improved infrastructure, and the role of better revenue generation on economic stability at the same time. This study contributes to the literature by addressing environmental sustainability and sustainable development as mediators between honest leadership, improved infrastructure, revenue generation, CPEC, and economic stability. Similarly, this study makes empirical implications as it guides the economic regulators to regulate and stabilize the economy by establishing honest leadership, applying improved infrastructure, and better revenue generation. These results also support one perspective of the Hegemonic stability theory that argues that international openness is the cause of common good.

## Conclusion

The results have proven that honest leadership in business enterprises has a positive association with economic stability. This paper examined that honest leadership in business enterprises improves enterprises’ performance, which is an excellent contribution to economic stability. The results prove that higher quality infrastructure brings stability in economic activities as quality infrastructure improves business organizations’ performance. The high rate of generation of revenue by business entities makes a positive contribution to economic stability. Moreover, it is concluded from the study that the CPEC project positively influences the economic stability of the associated countries. The introduction of the CPEC project between Pakistan and China has positively impacted the economy’s stability as it stimulates both countries’ economic activities. The CPEC has influenced different economic factors like leadership quality, the quality of infrastructure, and the revenue generation rate, which is of great importance in bringing stability to the economy.

While considering all the above, it is remarkably a fortuitous matter for us that despite the extreme pressure encountered by COVID-19 on almost every perspective of life. The CPEC not only sustains but also progresses under the leadership of all stakeholders. It is evident from the results of this research that when this projects works in its maximum capacity then it will support the economy of all stakeholders in a tremendous way despite the catastrophic effects caused by COVID-19.

### Research Limitation

Regardless of its theoretical and empirical contributions, this study has several limitations that future scholars should remove in their studies. The collection of data in support of this study has been collected through a particular source. Future academics should collect data for their studies from more than one source. Future scholars should make necessary changes in their studies to make them available and applicable across the world. Finally, honest leadership, improved infrastructure, and revenue generation affected the economic stability analyzed by the current study and suggested that future scholars should address many other factors that affect economic stability.

### Managerial Implication

Investors and managers must consider economic stability when making their decisions. This research helps them to understand the expected near future shifts in the business environment due to CPEC and prepare honest leaders with healthy infrastructure. Further, managers must consider that the government is focusing on economic stability with the help of environmental sustainability. Managers must bring themselves to the world standards of environmental protection which is also important during the COVID-19 pandemic.

## Data Availability Statement

The datasets presented in this article are not readily available because of the privacy and confidential nature of its respondents. Requests to access the datasets should be directed to waqas_sadiq2011@hotmail.com.

## Author Contributions

HL, JH, RK, and MS: conceptualization and writing – original draft. HL, JH, GA, and WA: data curation. HL, JH, and RK: formal analysis. GA, WA, MS, and JH: methodology. GA and WA: supervision. HL, JH, and MS: validation. HL, JH, RK, GA, WA, and MS: writing review and editing. All authors have read and approved the final version of the manuscript.

## Conflict of Interest

The authors declare that the research was conducted in the absence of any commercial or financial relationships that could be construed as a potential conflict of interest.
